# Efficacy and Safety of Acupuncture in Preterm and Term Infants

**DOI:** 10.1155/2013/739414

**Published:** 2013-06-13

**Authors:** Wolfgang Raith, Berndt Urlesberger, Georg M. Schmölzer

**Affiliations:** ^1^Division of Neonatology, Department of Paediatrics, Medical University of Graz, Auenbruggerplatz 30, 8036 Graz, Austria; ^2^Research Group for Paediatric Traditional Chinese Medicine, TCM Research Centre Graz (Acupuncture Research), Medical University of Graz, Auenbruggerplatz 30, 8036 Graz, Austria; ^3^Department of Pediatrics, University of Alberta, Edmonton, AB, Canada T6G 2R3; ^4^Department of Newborn Medicine, Neonatal Research Unit, Royal Alexandra Hospital, 10240 Kingsway Avenue NW, Edmonton, AB, Canada T5H 3V9

## Abstract

The aim of the paper was to review the literature about safety and efficiency of acupuncture therapy in term and preterm infants. We searched Medline, EMBASE, and Cochrane Central Register of Controlled Trials using a predefined algorithm, reviewed abstracts from the Pediatric Academic Society annual meetings (2000–2012), and performed a manual search of references in narrative and systematic reviews. A total of 26 studies identified met our search criteria. Only 6 of these studies met our inclusion criteria; however, two studies had to be excluded because the manuscripts were published in Chinese. Hence, only four studies were included in our analysis. Three of the four studies evaluated the effects of acupuncture on infantile colic, and one assessed pain reduction during minor painful procedures in preterm babies. The limited data available suggests that acupuncture could be a safe nonpharmacologic treatment option for pain reduction in term and preterm infants and could also be a non-pharmacologic treatment option to treat infantile colic. Currently acupuncture in infants should be limited to clinical trials and studies evaluating short- and long-term effects and should be performed only by practitioners with adequate training and experience in neonatal/pediatric acupuncture.

## 1. Introduction

Traditional Chinese Medicine (TCM) has been practiced in China for over 2000 years. TCM remained the main form of medical treatment within China before western medicine was introduced in the past 100 years. Traditional Chinese Medicine includes (i) massage therapy (=*Tuina*), (ii) moxibustion, (iii) and acupuncture. According to the available literature the pillars of pediatric treatment were massages, diets based on the five elements, and medicinal therapy. Observational studies reported that TCM is a popular treatment in children [1, 2]. In addition, there is increasing demand of complementary and alternative medicine (CAM) treatments in the pediatric population [3–7]. This trend towards CAM might be explained by dissatisfaction with conventional medicine as well as positive reports from friends and family [8–10].

There is a lack of data to support acupuncture, TCM, or CAM in children [11–14]. Only a few studies investigated the effect of acupuncture in children demonstrating positive effects on obesity [15], skin irritations, constipation, and pain [16, 17]. In addition, there is emerging evidence in individual cases of acupuncture in neonates [18] and to treat infantile colic [19–21]. Limitations of acupuncture in children are (i) their fear of needles and pain, which makes acupuncture difficult to perform in young children; (ii) infants or toddlers lacking cooperation, which makes the precise use of needle points challenging [22]; (iii) risk of infection caused by needle prick injuries [5, 23]; and (iv) safety of acupuncture being a major concern, particularly during early infancy when responses are difficult to evaluate.

Jindal et al. [24] reviewed the current evidence for acupuncture treatment including nausea and vomiting, asthma and seasonal allergic rhinitis, neurologic and gastrointestinal disorders, pain, and addiction. Overall, the most evidence for acupuncture comes from studies managing postoperative and chemotherapy-induced nausea/vomiting. Acupuncture seems to be most effective in preventing postoperative induced nausea in children. Acupuncture also appears to be well tolerated in children, has a low incidence of side effects, but fewer needles should be used when treating infants.

In general, acupuncture in children is usually limited to brief light needling using one-way needles, treatment by using acupressure, and giving mild stimulation and laser acupuncture.

The development of laser acupuncture allows new treatment options in children [25]. Laser acupuncture provides a noninvasive therapeutic approach, thus excluding the risk of infection caused by needle prick injuries [13, 24]. But the central and peripheral effects of laser acupuncture in infants have only been sporadic evaluated [26]. In particular the applied doses and the time of stimulation are a matter of ongoing discussions. However, there is increasing evidence from observational studies that acupuncture is a potential nonpharmacologic treatment for infants, term and preterm newborns, during their hospitalization in the intensive care unit [27, 28]. In particular newborn infants are exposed to sedative and analgesic medications, which are often prescribed for a prolonged period of time during their intensive care admission. Hence, the use of alternative or adjunctive comfort measures might decrease neonatal exposure to potentially neurotoxic agents. In addition, a pilot study by Golianu et al. [29] investigated the effect of acupuncture for the management of neonatal opioid and benzodiazepine withdrawal. The results are eagerly awaited. Also a study protocol for a Cochrane Review proposes to evaluate the effect of acupuncture in neonates with hypoxic ischemic encephalopathy compared with standard care but the details have never been published [30].

The aim of the paper was to review the literature about safety and efficiency of acupuncture therapy in term (between 37 and 42 weeks gestation), preterm infants (less 37 weeks gestation), and infants with infantile colic (infants within the 1st year of age).

## 2. Methods

### 2.1. Search Strategy

We searched Medline, EMBASE, and Cochrane Central Register of Controlled Trials using a predefined algorithm (the appendix), reviewed abstracts from the Pediatric Academic Societies annual meetings (2000–2012), and performed a manual search of references in narrative and systematic reviews. Discrepancies regarding inclusion were resolved through discussion among the review team.

### 2.2. Study Selection

Studies meeting the following criteria were included in the review: randomized control trial; comparing acupuncture versus placebo or versus medical treatment in preterm and term infants. The following outcomes were assessed: safety, efficiency, all-cause mortality, and death. Studies describing preterm infants were eligible if infants were born and treated <37 weeks gestation. Term infants were included if gestation age was between 37 and 42 weeks. In addition, studies investigating acupuncture during the neonatal period (day 1 to 28 after birth) were eligible. For infants with infantile colic studies were eligible for inclusion within the 1st year of age.

### 2.3. Data Extraction

Data were recorded using a standardized data collection form to record study design and methodological characteristics, patient characteristics, interventions, and outcomes, thereof, relative risk and 95% confidence interval (CI), as well as information regarding randomization mode, allocation concealment, blinding, and intention-to-treat analysis. Data extraction was independently performed by two investigators (GMS, WR) and discrepancies were resolved by consulting a third investigator (BU) through discussion.

### 2.4. Assessment of Methodological Quality

We assessed the methodological quality of the included trials and the risk of bias conferred by using elements of the Cochrane collaboration tool for assessing risk of bias [31]. The domains used in the present systematic review pertained to randomization and allocation concealment (selection bias), blinding (performance and detection bias), and adherence to the intention-to-treat principle (attrition bias). 

### 2.5. Statistical Analysis

**Figure 1:**
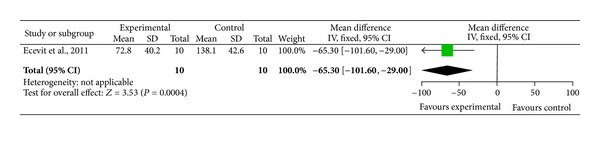
Forest plot of crying time for heel prick procedure in preterm infants with and without acupuncture.

We planned to measure the principal summary as weighted mean difference (WMD) for continuous outcomes, relative risk (RR), and the absolute risk reduction (RD) for dichotomous outcomes. For each trial, we planned to retrieve or calculate the crude RR and RD estimates and corresponding 95% CIs for the assessed outcomes. We planned to explore heterogeneity using a chi-square test and the quantity of heterogeneity using the *I* ^2^ [32] statistic [32]. We planned to summarize RR and RD estimates using random-effects models [33]. Analyses were performed in RevMan version 5 (Cochrane Collaboration, 2010). All *P* values are 2-tailed. We planned to calculate the numbers needed to treat (NNT) for all outcomes where the pooled estimates of RR were statistically significant. The study is reported according to the PRISMA checklist (Figure 1) [34].

## 3. Results

**Figure 2:**
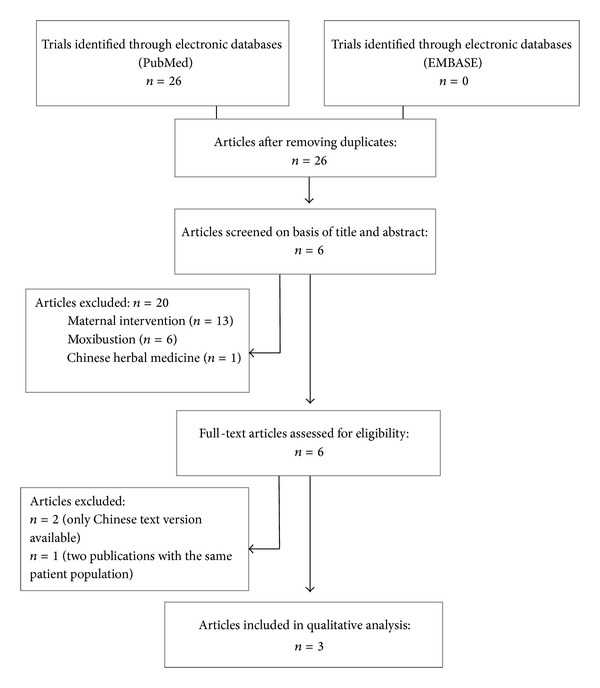
PRISMA flow chart.

A total of 26 studies identified met our search criteria (the appendix). However, 20 studies had to be excluded as they were evaluating the effect of acupuncture to resolve (i) breech presentation, (ii) mastitis during lactation, or (iii) pain during labor. Only 6 studies met our inclusion criteria (Figure 2); however, two further studies had to be excluded because the manuscripts were published in Chinese. Hence, only four studies were included in our analysis. Three of four studies evaluated the effects of acupuncture on infantile colic and one assessed pain reduction during minor painful procedures in preterm babies. In addition, we identified 28 abstracts from the Annual Meeting of the Pediatric Academic Societies addressing acupuncture in children. No abstract was identified reporting acupuncture in infants or newborn babies. 

Risk of bias assessment of included trials is presented in Table 1. We planned extensive statistical analysis; however, the trials identified (3x infantile colic, 1x pain in preterm infants) did not allow any of the planned analysis described in Section 2.5.

### 3.1. Pain in Preterm Infants

**Figure 3:**
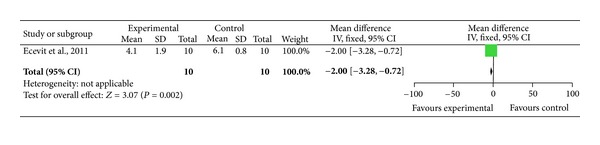
Forest plot of the neonatal infant pain scale score with and without acupuncture in preterm infants.

We identified one trial assessing the effect of acupuncture in preterm babies during minor painful procedures [28]. Using cross-over design 10 preterm infants were randomized to receive breast milk only or breast milk and acupuncture for a heel prick for blood gas analysis. Each infant acted as their own control and received either breast milk only on day one and on the following day breast milk and acupuncture or vice versa. Oxygen saturation, systolic and diastolic blood pressure, respiratory rate, and heart rate were similar before and after heel prick within groups. Crying duration (Figure 1) and neonatal infant pain scale scores (Figure 3) during heel prick were significantly lower in neonates who received acupuncture.

### 3.2. Infantile Colic

Two studies assessed crying [19, 20] and a third study assessed feeding, stooling, and sleeping patterns [35]. Overall a total of 121 infants were included to assess infantile colic.

Reinthal et al. quasirandomized 40 infants with excessive crying (median age: six weeks) to conventional or light needling treatment. Parents were blinded to the group assignment. Infants received acupuncture at the LI4 point on both hands for approximately 20 seconds on four occasions compared to conventional group. Parents had to complete pre- and posttreatment questionnaires to assess intensity, frequency, and duration of crying as well as pain-related behavior. Light needling resulted in a significant reduction in the rated crying intensity. Pain-related behavior like facial expression was also significantly less pronounced in the light needling group as compared to the control group. In addition, parents rated light needling as more effective in improving all symptoms than the control group. 

Landgren et al. [19] assessed the effect of acupuncture to reduce duration and intensity of crying in infantile colic. Eighty-one of the ninety included infants (2–8 weeks) completed a three weeks structured program consisting of six visits to an acupuncture clinic. Parents were blinded to the allocation of their children. Infants randomized to the treatment group received standardized acupuncture for 2 sec at the LI4 point in addition to standard of care. Infants randomized to acupuncture had a significant lower duration of fussing in the 1st (74 versus 129 min) and 2nd weeks (71 versus 102 min). In addition, a significant shorter duration of colicky crying in the 2nd intervention week (9 versus 13 min) was observed. In the same patients Landgren et al. [35] also assessed the infants sleep and stooling behavior (frequency and size). In addition, side effects were assessed using a parental questionnaire. Infants randomized to acupuncture had an increased stooling frequency compared to control group. No side effects were recorded. Overall, minimal needling acupuncture had no significant effect on feeding, stooling, or sleep. 

## 4. Discussion

### 4.1. Efficacy and Safety in Preterm Infants

One trial used breast milk compared to acupuncture and breast milk to assess pain during minor painful procedure in preterm infants [28]. Overall, the acupuncture was well tolerated, and the mean crying time (Figure 1) and the neonatal infant pain scale scores (Figure 3) were significantly lower in preterm infants receiving acupuncture compared to the control group. Although a significant reduction in crying time and pain score was observed in preterm infants receiving acupuncture, these results have to be interpreted with caution. The study by Ecevit et al. [28] had a very small sample size of 10 preterm infants using cross-over design. Light needling acupuncture was performed using acupuncture point Yintang (EXHN3), which is located midway between the medial ends of the eyebrows. Although a short and restful sleep and a significant decrease in heart rate during the procedure was observed, this might be due to the sedative effects of acupuncture point Yintang (EX-HN3) [28]. 

Further evidence about efficacy and safety in preterm infants comes from observational studies [23, 27]. Gentry et al. described 10 preterm and term infants receiving acupuncture in a retrospective chart review [27]. They observed a significant decrease in sedative and analgesic use in 5/8 infants treated with acupuncture therapy for agitation, over a time period of 2 weeks to 5 months [27]. Raith et al. described the first case report of laser acupuncture in a preterm infant [23]. After treatment a reduction in heart rate over time was observed [23]. In addition, acupuncture has been described for infantile cerebral palsy, neonatal stress and during hypoxic ischemic encephalopathy, and neonatal abstinence syndrome [18, 30, 36].

No study using needle acupuncture described any skin breakdown, infection, hematoma, or allergic reactions. In addition, no patient distress or discomfort was observed [27, 28]. Raith et al. [26] compared skin temperature before and 5 and 10 min after local laser needle acupuncture. On average an increase in local skin temperature of about 1°C was observed. In one case a maximum temperature of 37.9°C was observed [26]. However, the temperature increase was similar to transcutaneous CO_2_ measurements [37]. Furthermore, it remains unknown whether repeated needle stimulation may alter sensory processing and responses to subsequent painful stimuli, similar as heel pricks in infants, skin breakdown, or infection.

In summary current evidence suggests that acupuncture is feasible; however, more evidence is needed to determine efficacy and safety of this treatment in preterm and term infants. Only practitioners with adequate training and experience in neonatal/pediatric acupuncture should perform acupuncture treatments. 

### 4.2. Efficacy and Safety in Infants with Infantile Colic

Infantile colic is a common painful clinical condition associated with signs of distended intestines and an increase in colon peristalsis. We identified three studies evaluating acupuncture for the management of infantile colic [19, 20, 35]. All studies used the LI 4 (Hegu) point, which is considered to be one of the most effective acupuncture points for general pain control. In addition, it has been reported that LI4 interacts with serotonin and melatonin release and thereby with the circadian rhythm [38]. LI 4 is an acupuncture point in the large intestine meridian located on the radial side in the middle of the 2nd metacarpal bone. LI 4 is easily accessible and therefore easy to use in particular in young infants. The studies by Landgren et al. [19, 35] used short needling intervals of two seconds alternating between the right and left hands. The study by Reinthal et al. used “light needling” for 20 seconds bilaterally as minimal acupuncture technique [20]. Both trials reported a reduction crying frequency and intensity in the acupuncture group compared to controls. Limitations of Reinthal's study are (i) infants were older, which could have contributed to the remission rates, (ii) parents were blinded, but the same nurse who met the parents performed the acupuncture [20]. The main limitation from Landgren et al. [19] study is the increased crying incidence in the acupuncture group. Parents could have interpreted the crying as being in the treatment group, therefore providing a more positive feedback in the questionnaire. Overall, randomized trials reported that acupuncture reduced crying behavior of infants suffering from colic. Further evidence for efficacy and safety of acupuncture comes from observational studies [38, 39]. Reinthal et al. evaluated changes in gastrointestinal function after minimal acupuncture in 913 term infants at a mean age of 1.6 weeks [39]. Bilaterally light needling stimulation of LI4 was performed for 10–20 seconds daily [39]. Overall, frequency of regurgitation, belching, drooling, inflated stomach, and frequency of defecation decreased after treatment. In summary, acupuncture was well tolerated, safe, and with no serious side effects reported. Crying as a response to pain was the main side effect in the reported trials. Thirty-two of the 256 infants in the acupuncture group cried for more than 10 sec during the interventions compared to 14 infants in the control group. In addition, 37/256 infants cried >1 minute during acupuncture. Landgren et al. reported slight bleeding after needling in 1/256 acupuncture treatments in 81 randomized patients [19].

In summary current evidence suggests that acupuncture is safe, effective, and a cheap method to treat infantile colic [20].

### 4.3. Gaps of Knowledge to Treat Term and Preterm Infants

Currently acupuncture for term and preterm infants should be limited to clinical trials. Laser or needle acupuncture has been described [19, 23, 26–28, 35]; however, it remains unclear which treatment option is superior to treat preterm or term infants. Randomized trials should compare laser and needle acupuncture for the treatment of newborn infants. All included studies used a “light needling” technique. However, it remains unclear if a deeper needling technique would have yielded different results. The duration of acupuncture in the reported studies was very short, and a comparison of different acupuncture treatment duration is lacking. Acupuncture treatment is associated with a significant nocturnal increase in endogenous melatonin secretion and significant improvements in sleep onset latency, arousal index, total sleep time, and sleep efficiency [38]. Further studies are needed to clarify this relationship. In addition, clinical trials should focus on advantage, safety, and efficacy of acupuncture in the neonatal population. 

## 5. Conclusion

Acupuncture has the potential to decrease neonatal exposure to potentially neurotoxic analgesic and sedative agents during their early life. The limited data available suggests that acupuncture is a safe nonpharmacologic treatment option for pain reduction in term and preterm infants. However, no study has evaluated long-term effects of acupuncture. Currently acupuncture should be limited to clinical trials and studies evaluating short- and long-term effects are urgently needed.

## Figures and Tables

**Table 1:** Risk of bias assessment of randomized controlled trials investigating acupuncture in preterm and term infants.

Study	Study population	Comparison	Primary outcome measures	Sequence generation	Allocation concealment	Blinding of participants, personnel, and outcome	Incomplete outcome data	Selective outcome reporting	Funding bias
Ecevit et al. [28]	Preterm infants (*n* = 10)	Breast milk only or breast milk and acupuncture	Crying duration during heel prick for blood gas analysis	Unclear	Unclear	Unclear	Unclear	Unclear	Unclear
Landgren et al. [19, 35]	2–8 weeks old infants (*n* = 90)	Structured program versus structured program and needle acupuncture	Remission of infantile colic	Low	Low	Low	Low	Low	Low
Reinthal et al. [20]	Median 6 weeks old infants (*n* = 40)	Intervention versus control group	Crying intensity, frequency, duration of, crying and pain related behavior	Unclear	Unclear	Unclear	Low	Low	Low

## Appendix

*Search Strategies for PubMed (Last Search: February 14, 2013).* Limits activated: Humans, Randomized Controlled Trial, Clinical Trial (Phase I–IV), Child: birth to 18 years, Infant: birth to 23 months, Infant: 1–23 months, Newborn: birth to 1 month

1. MeSH descriptor *Infant*, explore all trees (Result: 40689)
2. MeSH descriptor *Newborn*, explore all trees (Result: 19274)
3. MeSH descriptor *Acupuncture*, explore all trees (Result: 714)
4. ((1) AND 2) AND 3 (Result: 26).

*Search Strategies for EMBASE (Last Search: February 14, 2013).* Limits activated: Humans, 1980 to current, Randomized Controlled Trial, Clinical Trial or controlled clinical trial, and (infant <to one year> or child <unspecified age

1. MeSH descriptor *Infant*, explore all trees (Result: 13023)
2. MeSH descriptor *Newborn*, explore all trees (Result: 4645)
3. MeSH descriptor *Acupuncture*, explore all trees (Result: 240)
4. ((1) AND 2) AND 3 (Result: 0).

## Abbreviations

TCM: Traditional chinese medicine
CAM: Complementary and alternative medicine
RCT: Randomized control trial
CI: Confidence interval
RR: Relative risk
RD: Absolute risk reduction
WMD: Weighted mean difference
NNT: Number needed to treat
HIE: Hypoxic ischemic encephalopathy
LI: Large intestine
LI4: Large intestine 4
GI: Gastrointestinal.
